# Development of an affective database made of interactive virtual environments

**DOI:** 10.1038/s41598-021-03380-y

**Published:** 2021-12-16

**Authors:** Nicolò Dozio, Federica Marcolin, Giulia Wally Scurati, Francesca Nonis, Luca Ulrich, Enrico Vezzetti, Francesco Ferrise

**Affiliations:** 1 Department of Mechanical Engineering, Politecnico di Milano, 20156 Milan, Italy; 2 Department of Management and Production Engineering, Politecnico di Torino, 10129 Turin, Italy

**Keywords:** Psychology, Emotion

## Abstract

Despite the great potential of Virtual Reality (VR) to arouse emotions, there are no VR affective databases available as it happens for pictures, videos, and sounds. In this paper, we describe the validation of ten affective interactive Virtual Environments (VEs) designed to be used in Virtual Reality. These environments are related to five emotions. The testing phase included using two different experimental setups to deliver the overall experience. The setup did not include any immersive VR technology, because of the ongoing COVID-19 pandemic, but the VEs were designed to run on stereoscopic visual displays. We collected measures related to the participants’ emotional experience based on six discrete emotional categories plus neutrality and we included an assessment of the sense of presence related to the different experiences. The results showed how the scenarios can be differentiated according to the emotion aroused. Finally, the comparison between the two experimental setups demonstrated high reliability of the experience and strong adaptability of the scenarios to different contexts of use.

## Introduction

The attention towards human emotions has been growing in recent years, given the importance they have on different aspects of everyday life. Emotions affect how we face and react to any experience, event, and situation. Despite there is not a univocal definition of “emotion”, the psychology and neuroscience fields have widely acknowledged that emotions are deeply related to human health, well-being, behaviour, thinking, and decision-making, impacting the personal and social dimension of an individual^1^. Therefore, arousing emotional reactions through validated stimuli can have different application interests, and the demand is greater than in the past.

Among the various media technologies that can elicit emotional reactions, one of the most interesting is Virtual Reality (VR). Through VR, we can provide users with immersive experiences delivering a variety of sensory stimuli. As a result, users can truly feel to be in the virtual environment (i.e., what is usually defined as the sense of presence^2–4^) and to control and interact effectively with it (i.e., the sense of agency^5, 6^). Furthermore, these aspects can strongly contribute to eliciting specific emotions in a way that is comparable and not significantly different from real-life experiences^7^.

Interactions between the level of presence and the emotional experience have been consistently reported in the literature^8, 9^. Despite there is not total agreement on the nature of this interaction, there seems to be also a link between the level of immersion experienced in the VR environment and the emotional response, especially in terms of arousal, one of the commonly used dimensions to describe the emotional reaction^9^. VR environments’ ease of interaction leads to the opportunity of inducing relaxations states supporting stress recovery^10^ or to support the development of sustainable attitudes and behaviours^11^. Furthermore, VR can also enhance our ability to empathize with others^12^. However, this capability can also emerge as a side effect, as the strengthening effect of VR on emotions is not limited to positive experiences. Indeed, it also includes negative emotions, for instance, comparing the effects of VR to PC games^13^. Hence, regardless of the intentional use of emotions to achieve specific goals, VR designers should always consider and manage emotional aspects. For this reason, there is a need to understand how to generate, or rather avoid triggering, certain emotions through VR. The study of human emotions’ elicitation through specific stimuli has a long tradition, and guidelines and methodologies regarding aesthetic features and attributes (e.g.,^14, 15^) have been developed to support the design of emotional artefacts.

The International Affective Picture System (IAPS)^16^ still constitutes one of the most used tools for inducing emotions. Other images databases are the Geneva affective picture database (GAPED)^17^, the EmoPicS database^18^, and the Nencki Affective Picture System (NAPS)^19^, developed to provide wider sets of increasing quality, realistic pictures. The extensive utilization of static image databases is probably due to the ease of use of this media, which provide to experimenters a high level of control on the presentation methods and requires a relatively low demand in terms of cognitive resources to participants, making it usable for a great variety of populations. However, despite their diffusion, static images have some limitations, primarily linked with their ecological validity and the intensity of the emotion elicited. The relationship between sound and emotions has also been widely explored^20^ and affective databases are available also for this sensory modality (e.g. IADS^21^). The study of auditory stimuli includes verbal and non-verbal sounds, as well as music. A set of descriptors suitable to evaluate all of them is presented by Weninger et al.^22^. Words’ phonetic features can make them sound pleasant or harsh, influencing their affective rating and also their meaning^23^. The Oxford Vocal (OxVoc) is a database of non-verbal sounds performed by adults, children, and animals emitting sad, happy, and neutral vocal expressions^24^. Considering music, fast tempo and major mode are linked to positive and active emotions, while slow tempo and minor mode are associated with negative and passive ones^25^. To overcome unisensory limitations, in the 90s, Gross et al.^26^ developed the first movies affective database, made up of sixteen films eliciting amusement, anger, contentment, disgust, sadness, surprise, neutrality, and, slightly less successfully, fear. Their work has been followed by more recent studies (e.g.^27–29^), which also differ according to the elicited emotional states. Movie clips surely offer a more involving experience than static images, however they are generally passive. The viewer does not control or interact with the presented events; similar limitations can be overcame using VR tools. Despite the use of emotional and affective media content has led to the creation of several databases of images, sounds, and videos, today and to the best of our knowledge, there are still no databases or design guidelines considering VR experiences and environments raising emotional responses.

In recent years, there also have been attempts to create affective databases through 360 °C video clips. In the work conducted by Li et al.^30^, 73 selected video clips have been rated on the Valence and Arousal dimensions using the Self-Assessment Manikin scale, finding a correlation between valence ratings and standard deviation of head yaw. The video clips were played using a Head-Mounted Display (HMD) providing a good level of immersion and allowing experimenters to track head movements. Similarly, Jun et al.^31^ used HMD and immersive video clips to collect, among others, data related to presence, arousal, and simulator sickness on a large participants sample. Nevertheless, the contents considered in these works were video clips selected by the authors from the public domain and did not allow participants to interact with the content presented.

Virtual environments could represent the most effective way to arouse emotions because they exploit the benefits of being multisensory and include the possibility of interaction, which is an important element for users’ involvement. The novelty and the purpose of the current work are to provide a database of validated interactive virtual environments (VEs) related to five distinct emotional categories to be used for the development of affective VR experiences. We followed a categorization based on five emotions (i.e. anger, fear, disgust, sadness, and happiness) considered to be shared universally, meaning-wise^32^. The ten newly developed affective VEs were tested on two experimental setups (Exp. 1 and Exp. 2), differentiated by the technology used to deliver the virtual experience. At this stage, none of the experimental set up was based on immersive VR technologies due to the ongoing COVID-19 pandemic. However, we can define the interactive VEs as scenarios that can be navigated by the users and respond to their movements into the virtual space. For this reason, they can easily be exported for other platforms, in particular for VR headsets. The comparison showed a consistency in the results, thus proving that the environments work independently of the rendering technology.

## Methods

The study was conducted simultaneously in two experimental setups (i.e., Exp. 1 and Exp. 2) differentiated by the technology used to render the VEs. The experiments followed a within-subjects design where the five emotional entities used to categorise our affective VEs, plus neutrality and the baseline, constituted our experimental conditions.

### Participants

The study involved a total of 75 participants. who voluntary took part to the experiment. In particular:

- Exp. 1 involved 48 participants (17 female), aged between 19 and 40 years (*M* = 26,27; *SD* = 4336).
- Exp. 2 involved 27 participants (14 female), aged between 19 and 34 years (*M* = 24,52; *SD* = 3378).

For both the experimental setups, each participant provided written informed consent for study participation. Written consent and all methods were carried out in accordance with the principles of the Helsinki Declaration.

### Affective VEs

We used eleven VEs for the experiment. Ten scenarios, two for each of the five basic emotions (i.e. anger, fear, disgust, sadness, happiness), are newly developed. The additional scenario, for neutrality, is described in^33^.

**Figure 1:**
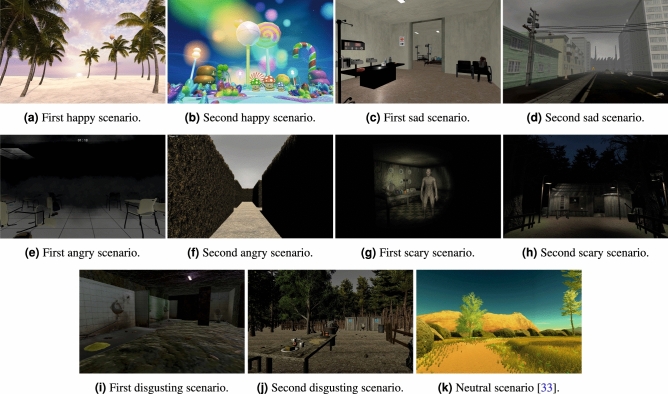
Details of the affective VR environments used to elicit Happiness, Sadness, Anger, Fear, Disgust, and Neutrality.

*Happy scenarios* The happiness scenarios were designed differently since one aimed at creating happiness through relaxation and the other through fun and excitement. Both the scenes have a positive valence, but arousal is variable. The first scene consisted of a sunset on a tropical beach with palm trees; the user has to walk towards and into the calm sea (Fig. 1a). The second scene featured a fantastic rainbow world with colourful bubble lights and funny animals and vegetation (Fig. 1b).

*Sad scenarios* The first consisted of an abandoned hospital, with people suffering and crying (Fig. 1c). The second was a post-apocalyptic scene, with industrial buildings, dead animals and vegetation (Fig. 1d).

*Angry scenarios* The first was a school, during a fire, with smoke preventing users from seeing the surrounding clearly (Fig. 1e). The second was a labyrinth garden with high fences (Fig. 1f). In both scenes, users have to find the exit, but invisible obstacles lead them to start the game repeatedly, showing a red flashing light. Countdowns stimulate users to move quickly.

*Scary scenarios* The first consisted of a dark, haunted house, with monsters, clowns and typical horror movie events (e.g., TV turning on, door shutting) (Fig. 1g). The second featured a dark wood populated by monsters, with dead bodies and zombies (Fig. 1h).

*Disgusting scenarios* The first represented an abandoned basement, with insects, rats and dirty toilets (Fig. 1i). The second was a picnic site, with dirty dishes and rotten food, insects, mud, and chemical toilets with people vomiting (Fig. 1j).

*Neutral scenario* We used the neutral environment developed by Chirico et al.^33^ which was constituted by a circular path located in a small meadow surrounded by rocks and a few trees (Fig. 1k). Auditory features were excluded in this scenario to limit any possible emotional influence further.

### Measures

#### Empathy

Before taking part in the study, participants were asked to fill in a questionnaire that aimed to assess their empathy level. We used an Italian translated and validated version of the Balanced Emotional Empathy Scale (BEES)^34^.

#### Emotion ratings

After each scenario presentation, participants were asked to rate on seven continuous scales from 0 to 100 to measure the intensity with which the participants’ associated emotional labels corresponding to 6 basic emotions^32^, plus neutrality, with the presented scenario. The labels associated with the scales were happiness, sadness, anger, fear, disgust, surprise, and neutrality.

#### Presence

Presence measures were collected using Version 3.0 of the Presence Questionnaire^35^. The questionnaire uses a 7-point scale format where each item is anchored at the ends by opposing descriptors, while in the middle, a midpoint anchor is shown. This version of the questionnaire is an update of the Version 2.0^3^ and present a decrease in the number of items from 32 to 29 items of the Version 3.0 (by removing item 26, 27 and 28) and provide a new four factors structure named Involvement (item 1, 2, 3, 4, 6, 7, 8, 10, 14, 17, 18, 29), Sensory fidelity (item 5, 11, 12, 13, 15, 16), Adaptation/immersion (item 9, 20, 21, 24, 25, 30, 31, 32), and Interface quality (item 19, 22, 23).

The numbering of the Version 3.0 items reflects the original numbering of Version 2.0 to which items 26, 27 and 28 have been removed.

Since some of the items were referred to the assessment of tactile/haptic feedbacks (i.e. item 13, 17, 29) or focused on measuring specific interaction dynamics or activities which were not feasible nor requested during our exploration tasks (i.e. items 7, 9, 15, 16, 23, 24, 31), we decided to exclude them from data collection, resulting in an adapted version of the questionnaire constituted by 19 items. We used Cronbach’s alpha to measure the reliability of our modified 19-item scale, which was still acceptable (*alpha* = 0.83; N = 336). We also computed internal consistency reliability coefficients for the original four factors with the remaining 19 items:

- Involvement (item 1, 2, 3, 4, 6, 8, 10, 14, 18) (*alpha* = 0.82);
- Sensory fidelity (item 5, 11, 12) (*alpha* = 0.83);
- Adaptation/immersion (item 20, 21, 25, 30, 32) (*alpha* = 0.65);
- Interface quality (item 19, 22) (*alpha* = 0.47).

### Procedure

The testing was performed simultaneously in two setups, differentiated by the technology used to render the virtual environments. Before the scenario presentation, participants were requested to read and sign a consent form. Then, they were instructed about the structure of the study and the composition of the questionnaires. After the explanation, they were asked to first assess their current emotional state on the seven emotional labels, which constituted our baseline. We then gave them a mouse and a keyboard. We proceeded with a brief training session on a simple virtual environment developed with Unity Software (version 2019.4.13f1), constituted by a floor and some geometric 3D obstacle. Participants controlled their movements with WASD keys and oriented their point of view with the mouse. When they felt confident with the controls, participants communicated that they were ready to start the exploration session, in which they were asked to navigate and explore seven scenarios, one for each discrete emotion plus the neutral scenario. Each scene had a fixed duration of 90 seconds. Standardised scenario selection and presentation order was counterbalanced between participants using a Latin Square design. After each scenario, participants were asked to rate the seven emotional labels and complete our adapted version of the Presence Questionnaire. In Exp. 2, Presence measures were collected one time at the end of the experiment. The assessment phase between the presentation of the scenarios lasted 5 min on average and this break, combined with the order of the counterbalanced scenarios, helped us counteract any residual emotional bias between different conditions. The entire experimental session lasted about 45 min. All data were analysed and reported anonymously.

### Experimental setups

*Exp. 1:* participants were asked to sit on a fixed chair located in front of a wall screen (width 3; height 2.5 meters) at a distance of 2 m from the projected wall. VR environments were projected using a projector (resolution 1920 × 1080p; framerate 60 fps) connected to a PC (GPU: NVIDIA GTX 2070 Max-Q; CPU: Intel i7; RAM 32 GB). The audio was played through over-ear headphones connected to the PC., The use of HMDs was discarded due to the general laboratory safety practices during the COVID-19 Pandemic.

*Exp. 2:* participants were seated on a fixed chair located in front of a desktop computer at a distance of 70 cm from it. VR scenarios were displayed on a monitor (screen size: 27 inches; resolution: 2560 × 1440) connected to a PC (GPU: NVIDIA QUADRO P3200; CPU: Intel(R) Xeon(R) E-2186M @ 2.90GHz; RAM: 32 GB). Audio was played through a speaker connected to the PC., In this experiment an RGB-D camera was added to capture facial expressions for future analyses on Facial Expression Recognition (FER). The use of HMDs was discarded in order to reduce occlusion issues and because of the general laboratory safety practices during the COVID-19 Pandemic.

Though head tracking technology might have improved interaction with VEs, we decided not to use it. The reason is that in Exp. 1, participants were asked to sit on a chair. Thus head position was supposed not to change significantly. About head orientation, usually in using big wall displays, rotations are removed, and only displacements are left to track. This is because the user is supposed to look at the unique available wall to visualize the information. In Exp. 2, we included an RGB-D camera, and the user was supposed not to move or rotate the head to simplify data acquisition.

### Data analysis

For the empathy score, we performed a mixed repeated measures ANOVA with our experimental conditions as within-subjects factor, the emotional labels as measures and the questionnaire score as a between-subjects variable.

Two normality tests (i.e. Kolmogorov-Smirnov, Shapiro-Wilk) were carried out to determine if data related to the emotional labels were normally distributed. They did not follow a normal distribution, so we decided to proceed by using a non-parametric Friedman test analysis of differences among repeated measures. Then, a post hoc analysis with Wilcoxon signed-rank test was conducted. Since multiple comparisons increase the probability to commit Type I errors, a Bonferroni correction was applied to lower the critical p-value depending on the number of tests performed. We had 7 conditions (including baseline) resulting in 21 (= 7 × 6/2) [N(N-1)/2] possible combination, therefore we adjusted the significance level to 0.002 (= 0.05/21)^36^.

For Presence rates, we performed two normality test (i.e. Kolmogorov–Smirnov and Shapiro–Wilk), and the results showed that the data related to the four factors of the Presence Questionnaire were normally distributed. We, therefore, proceeded with one-way repeated measures ANOVAs (conditions: Neutral, Happy, Sad, Scary, Angry, Disgusting) with our four factors as measures (i.e. Involvement, Sensory fidelity, Adaptation/Immersion, and Interface quality). We then performed post hoc comparisons using the Bonferroni correction (*alpha* = 0.05).

Regarding the comparison between the two setups, data related to the emotional labels were not normally distributed. Therefore, we performed a Mann-Withney U test for each emotional label to compare the results obtained in our experimental conditions among the two different setups.

Finally, to compare the Presence scores between the two experimental setups, we computed an overall Presence score for Exp. 1 using the data collected for each condition. We then performed a one-way ANOVA to compare the overall Presence score and the four constitutive factors (i.e., Involvement, Sensory fidelity, Adaptation/immersion, Interface quality) between the two setups.

## Results

### Empathy

We collected 67 responses ($N_{Exp.1}$ = 43; $N_{Exp.2}$ = 24). Empathy scores were classified in three different categories: above, below, and corresponding to the average of the population in which the questionnaires were validated. Responses showed that 19 participants in Exp. 1 and 12 participants in Exp. 2 obtained a lower score than the mean of the population.

*Exp. 1:* Results showed an interaction effect of empathy scores and conditions on Sadness [*F*(6.696) = 3.571; *p* $< 0.001$; $\eta _{p}^{2}$ = 0.155] and Anger [*F*(7.089) = 2.910; *p* = 0.007; $\eta _{p}^{2}$ = 0.130] rates.

In particular, participants with a lower level of empathy reported lower Sadness rates in the Sad (*M* = 26.32; *SD* = 20.02) and Angry (*M* = 4.84; *SD* = 8.35) conditions, than those with empathy scores corresponding to the average (Sad condition: *M* = 49.18; *SD* = 28.21) or above the average (Sad condition: *M* = 57.67; *SD* = 25.54; Angry condition: *M* = 23.83; *SD* = 30.02). Furthermore, in the Scary condition empathy scores above the average corresponded with higher rates of Anger (*M* = 39,0; *SD* = 37.27) than those with an average (*M* = 12.41; *SD* = 16.56) or below the average (*M* = 7.79; *SD* = 13.94) score at the BEES questionnaire.

*Exp. 2:* Results did not show a significant effect (*p* $> 0.05$) of BEES scores on the seven emotional scales in our experimental conditions.

### Emotion ratings

Participants were asked to rate on seven continuous scales from 0 to 100 the intensity with which they experienced six basic emotions^32^, plus neutrality, according to the presented scenario. The labels associated with the scales were happiness, sadness, anger, fear, disgust, surprise, and neutrality. The experiments followed a within-subjects design where the five emotional entities used to categorised our VR scenarios, plus the neutral scenario and the baseline, constituted our experimental conditions. The results showed a statistically significant difference for each scale depending on which scenario was presented (i.e., the experimental condition). More specifically:

- Happiness—Exp. 1: ${\chi }^2$(6) = 147.382; *p* $< 0.001$. Exp. 2: ${\chi }^2$(6) = 107.698; *p* $< 0.001$.
- Sadness—Exp. 1: ${\chi }^2$(6) = 120.835; *p* $< 0.001$. Exp. 2: ${\chi }^2$(6) = 52.590; *p* $< 0.001$.
- Anger—Exp. 1: ${\chi }^2$(6) = 127.306; *p* $< 0.001$. Exp. 2: ${\chi }^2$(6) = 63.215; *p* $< 0.001$.
- Fear—Exp. 1: ${\chi }^2$(6) = 159.074; *p* $< 0.001$. Exp. 2: ${\chi }^2$(6) = 68,945; *p* $< 0.001$.
- Disgust—Exp. 1: ${\chi }^2$(6) = 175.720; *p* $< 0.001$. Exp. 2: ${\chi }^2$(6) = 101.449; *p* $< 0.001$.
- Surprise—Exp. 1: ${\chi }^2$(6) = 44.682; *p* $< 0.001$. Exp. 2: ${\chi }^2$(6) = 58.931; *p* $< 0.001$.
- Neutrality—Exp. 1: ${\chi }^2$(6) = 86.193; *p* $< 0.001$. Exp. 2: ${\chi }^2$(6) = 26.848; *p* $< 0.001$.

**Figure 2:**
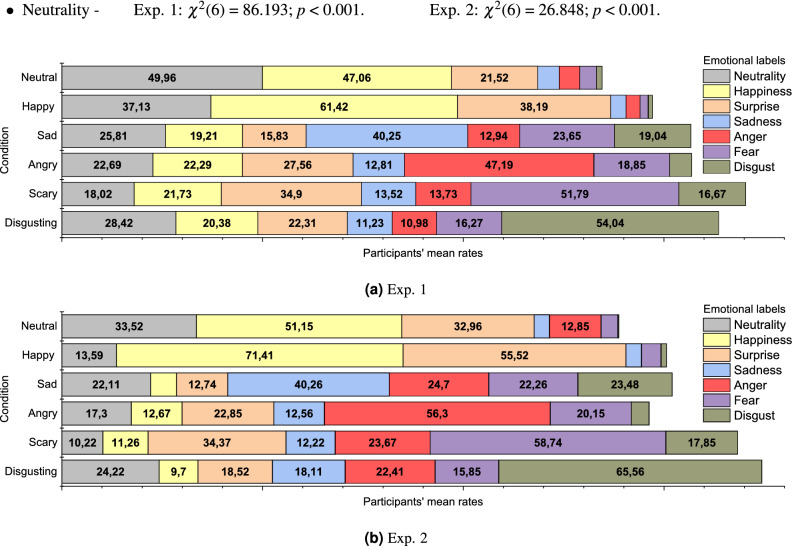
Participants’ mean rates for the 7 emotional labels in the two experimental setups.

Figure 2a and b show participants’ rates in our two experimental setups, while Tables 1 and 2 show the results for each emotional label in the condition in which it obtained the highest rating, compared to all the other conditions (e.g., participants’ rates for the label *‘Anger’* in the Angry condition compared to the rates for the same label in all the other conditions).

**Table 1:** Exp. 1—comparison of each label in the condition with the highest rating and all other conditions.

VS	Baseline		Neutral		Happy		Sad		Angry		Scary		Disgusting	
*Condition/label*	*Z*	*p*	*Z*	*p*	*Z*	*p*	*Z*	*p*	*Z*	*p*	*Z*	*p*	*Z*	*p*
Neutral/neutrality	− 0.807	0.42			− 3.162	0.002	− 4.566	<0.001	− 4.717	<0.001	− 5.062	<0.001	− 4.344	<0.001
Happy/happiness	− 0.692	0.489	−4.217	<0.001			− 5.563	<0.001	− 5.715	<0.001	− 5.480	<0.001	− 5.469	<0.001
Happy/surprise	− 3.222	0.001	− 3.616	<0.001			− 4.797	<0.001	− 2.290	0.022	− 1.002	0.316	− 3.474	0.001
Sad/sadness	− 5.468	<0.001	− 5.647	<0.001	− 5.647	<0.001			− 5.647	<0.001	− 5.489	<0.001	− 5.393	<0.001
Angry/anger	− 5.836	<0.001	− 5.597	<0.001	− 5.844	<0.001	− 5.779	<0.001			− 5.580	<0.001	− 5.532	<0.001
Scary/fear	− 5.804	<0.001	− 5.907	<0.001	− 5.907	<0.001	− 5.907	<0.001	− 5.443	<0.001			− 5.751	<0.001
Disgusting/disgust	− 5.876	<0.001	− 5.779	<0.001	− 5.843	<0.001	− 5.907	<0.001	− 5.730	<0.001	− 5.519	<0.001

**Table 2:** Exp. 2—comparison of each label in the condition with the highest rating and all other conditions.

VS	Baseline		Neutral		Happy		Sad		Angry		Scary		Disgusting	
*Condition/label*	*Z*	*p*	*Z*	*p*	*Z*	*p*	*Z*	*p*	*Z*	*p*	*Z*	*p*	*Z*	*p*
Neutral/neutrality	− 0.943	0.345			− 2.810	0.005	− 1.429	0.153	− 1.582	0.114	− 2.552	0.011	− 1.289	0.197
Happy/happiness	− 2.845	0.004	− 3.392	0.001			− 4.544	<0.001	− 4.383	<0.001	− 4.471	<0.001	− 4.459	<0.001
Happy/surprise	− 4.004	<0.001	− 3.418	<0.001			− 4.203	<0.001	− 3.499	<0.001	− 2.775	0.006	− 4.230	<0.001
Sad/sadness	− 3.608	<0.001	− 4.386	<0.001	− 4.203	<0.001			− 3.813	<0.001	− 3.863	<0.001	− 3.318	0.001
Angry/anger	− 3.634	<0.001	− 4.244	<0.001	− 4.375	<0.001	− 3.378	0.001			− 3.149	0.002	− 3.652	<0.001
Scary/fear	− 3.846	<0.001	− 4.461	<0.001	− 4.545	<0.001	− 4.258	<0.001	− 4.295	<0.001			− 4.214	<0.001
Disgusting/disgust	− 4.172	<0.001	− 4.547	<0.001	− 4.545	<0.001	− 4.495	<0.001	− 4.545	<0.001	− 4.321	<0.001		

#### Neutrality

In *Exp. 1*, Neutrality rates in the Neutral condition (*M* = 49.96) were significantly higher than the Happy (*M* = 37.13; *Z* = − 3.162; *p* = 0.002), Sad (*M* = 25.81; *Z* = − 4.566; *p* $< 0.001$), Angry (*M* = 22.69; *Z* = − 4.717; *p* $< 0.001$), Scary (*M* = 18.02; *Z* = − 5.062; *p* $< 0.001$), and Disgusting (*M* = 28.42; *Z* = − 4.344; *p* $< 0.001$) conditions. Neutrality rates did not differ significantly from the Baseline condition (*M* = 55.69; *Z* = − 0.807; *p* = 0.42).

In *Exp. 2*, Neutrality rates in the Neutral condition (*M* = 33.52) did not differ significantly (*p* $\ge$ 0.002) from any of the other experimental conditions.

#### Happiness

In *Exp. 1*, Happiness rates in the Happy condition (*M* = 61.42) were significantly higher than the Neutral (*M* = 47.06; *Z* = − 4.217; *p* $< 0.001$), Sad (*M* = 19.21; *Z* = − 5.563; *p* $< 0.001$), Angry (*M* = 22.29; *Z* = − 5.715; *p* $< 0.001$), Scary (*M* = 21.73; *Z* = − 5.480; *p* $< 0.001$) and Disgusting (*M* = 20.38; *Z* = − 5.469; *p* $< 0.001$) conditions.

In *Exp. 2*, Happiness rates in the Happy condition (*M* = 71.41) were significantly higher than the Neutral (*M* = 53.19; *Z* = − 3.392; *p* = 0.001), Sad (*M* = 6.48; *Z* = − 4.544; *p* $< 0.001$), Angry (*M* = 12.67; *Z* = − 4.383; *p* $< 0.001$), Scary (*M* = 11.26; *Z* = − 4.471; *p* $< 0.001$) and Disgusting (*M* = 9.7; *Z* = − 4.459; *p* $< 0.001$) conditions.

#### Surprise

In *Exp. 1*, Surprise rates were highest in the Happy condition (*M* = 38.19) and they differed significantly from the Baseline (*M* = 21.94; *Z* = − 3.222; *p* = 0.001), Neutral (*M* = 21.52; *Z* = − 3.616; *p* $< 0.001$), Sad (*M* = 15.83; *Z* = − 4.797; *p* $< 0.001$) and Disgusting (*M* = 22.31; *Z* = − 3.474; *p* = 0.001) conditions.

In *Exp. 2*, Surprise rates were highest in the Happy condition (*M* = 55.54) and they differed from all the other conditions except the Scary one (*M* = 34.37; *Z* = − 2.775; *p* = 0.006).

#### Sadness

In both Exp. 1 and Exp. 2, Sadness rates in the Sad condition ($M_{Exp.1}$ = 40.25; $M_{Exp.2}$ = 40.26) were significantly higher (*p* $< 0.001$) than all the other conditions.

*Exp. 1:* Baseline (*M* = 6.92; *Z* = − 5.468), Neutral (*M* = 5.35; *Z* = − 5.647), Happy (*M* = 3.79; *Z* = − 5.647), Angry (*M* = 12.81; *Z* = − 5.647), Scary (*M* = 13.52; *Z* = − 5.489), and Disgusting (*M* = 11.23; *Z* = − 5.393).

*Exp. 2:* Baseline (*M* = 14.37; *Z* = − 3.608), Neutral (*M* = 3.81; *Z* = − 4.386), Happy (*M* = 3.81; *Z* = − 4.203), Angry (*M* = 12.56; *Z* = − 3.813), Scary (*M* = 12.22; *Z* = − 3.863), and Disgusting (*M* = 18.11; *Z* = − 3.318).

#### Anger

In both the experimental setups, Anger rates in the Angry condition ($M_{Exp.1}$ = 47.19; $M_{Exp.2}$ = 56.3) were significantly higher (*p* $< 0.001$) than all the other conditions.

*Exp. 1:* Baseline (*M* = 5.17; *Z* = − 5.836), Neutral (*M* = 5.13; *Z* = − 5.597), Happy (*M* = 3.54; *Z* = − 5.844), Sad (*M* = 12.94; *Z* = − 5.358), Scary (*M* = 13.73; *Z* = − 5.580), and Disgusting (*M* = 10.98; *Z* = − 5.532).

*Exp. 2:* Baseline (*M* = 17.85; *Z* = − 3.634), Neutral (*M* = 12.85; *Z* = − 4.244), Happy (*M* = 0.07; *Z* = − 4.375), Sad (*M* = 24.7; *Z* = − 3.378), Scary (*M* = 23.67; *Z* = − 3.149; *p* = 0.002), and Disgusting (*M* = 22.41; *Z* = − 3.652).

#### Fear

In both Exp. 1 and Exp. 2, Fear rates in the Scary condition ($M_{Exp.1}$ = 51.79; $M_{Exp.2}$ = 58.74) were significantly higher (*p* $< 0.001$) than all the other conditions.

*Exp. 1:* Baseline (*M* = 6.40; *Z* = − 5.804), Neutral (*M* = 4.21; *Z* = − 5.907), Happy (*M* = 2.00; *Z* = − 5.907), Sad (*M* = 23.65; *Z* = − 5.443), Angry (*M* = 13.73; *Z* = − 5.508), and Disgusting (*M* = 16.27; *Z* = − 5.751).

*Exp. 2:* Baseline (*M* = 14.37; *Z* = − 3.846), Neutral (*M* = 4.22; *Z* = − 4.461), Happy (*M* = 4.85; *Z* = − 4.545), Sad (*M* = 22.26; *Z* = − 4.258), Angry (*M* = 20.15; *Z* = − 4.295), and Disgusting (*M* = 15.85; *Z* = − 4.214).

#### Disgust

For both the experimental setups, Disgust rates in the Disgusting condition ($M_{Exp.1}$ = 54.04; $M_{Exp.2}$ = 65.56) were significantly higher (*p* $< 0.001$) than all the other conditions.

*Exp. 1:* Baseline (*M* = 1.33; *Z* = − 5.876), Neutral (*M* = 1.35; *Z* = − 5.779), Happy (*M* = 1.08; *Z* = − 5.843), Sad (*M* = 19.04; *Z* = − 5.730), Angry (*M* = 5.50; *Z* = − 5.519), and Scary (*M* = 16.67; *Z* = − 5.523).

*Exp. 2:* Baseline (*M* = 12.11; *Z* = − 4.172), Neutral (*M* = 0.22; *Z* = − 4.547), Happy (*M* = 1.37; *Z* = − 4.545), Sad (*M* = 23.48; *Z* = − 4.495), Scary (*M* = 17.85; *Z* = − 4.321), and Angry (*M* = 4.48; *Z* = − 4.545).

### Presence

**Figure 3:**
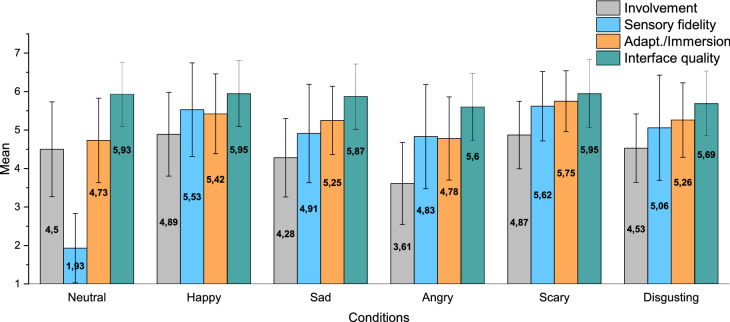
Exp. 1—presence mean rates for each experimental condition.

Participants were asked to fill our adapted version of the presence questionnaire after each condition only during Exp. 1. For this reason, the following analysis will consider only data from Exp. 1, while data from Exp. 2 will be considered only for a comparison between the two setups. Presence mean rates for each condition are shown in Fig. 3.

#### Involvement

We observed a significant effect of conditions on Involvement [*F*(5) = 18.927; *p* $< 0.001$; $\eta _{p}^{2}$ = 0.287]. As visible from Fig. 3, the less involving condition was Angry (*M* = 3.61; *SD* = 1.065) which differed significantly (*p* $< 0.05$) from all the other conditions. Furthermore, the Happy (*M* = 4.89; *SD* = 1.087) and Scary (*M* = 4.87; *SD* = 0.876) conditions also had a significant higher level of involvement compared to the Sad condition (*M* = 4.28; *SD* = 1.02).

#### Sensory fidelity

Results showed a significant effect of the experimental conditions on Sensory Fidelity [*F*(5) = 76.644; *p* $< 0.001$; $\eta _{p}^{2}$ = 0.620]. The Neutral condition (*M* = 1.93; *SD* = 0.9) was significantly lower than all the other conditions, but since the items in this factor were mainly related to auditory features, which were voluntarily absent in our Neutral scenario, we expected to obtain lower results. Pairwise comparison also shows a significant higher level for sensory fidelity in Happy (*M* = 5.53; *SD* = 1.218) and Scary (*M* = 5.62; *SD* = 0.903) conditions compared to Sad (*M* = 4.91; *SD* = 1.277), and finally a significant difference between Scary and Angry (*M* = 4.83; *SD* = 1.081).

#### Adaptation/immersion

Conditions had a significant effect on Adaptation/Immersion [*F*(4.175) = 14.369; *p* $< 0.001$; $\eta _{p}^{2}$ = 0.234]. Also for this factor, the Neutral condition (*M* = 4.73; *SD* = 1.099) had a significantly lower mean compared to all the other conditions, except for Angry (*M* = 4.78; *SD* = 1.081), which differed significantly only from Happy (*M* = 5.42; *SD* = 1.099), and Scary (*M* = 5.75; *SD* = 0.791) conditions. The highest level of Adaptation/Immersion was reached by the Scary condition, which was also significantly higher than Sad (*M* = 5.25; *SD* = 0.887) and Disgusting (*M* = 5.26; *SD* = 0.969) conditions.

#### Interface quality

Result showed a small effect of conditions on Interface quality [*F*(3.974) = 2.455; *p* $< 0.05$; $\eta _{p}^{2}$ = 0.05]. Pairwise comparisons only showed an almost significant difference (*p*= 0.052) between the Scary *M* = 5.95; *SD* = 0.883) and the Angry *M* = 5.6; *SD* = 0.875) conditions.

### Comparison between Exp. 1 and Exp. 2

In this section, we compare the results obtained in the two experimental setups to explore effects linked to the differences in the presentation of our scenarios.

#### Emotion ratings

**Figure 4:**
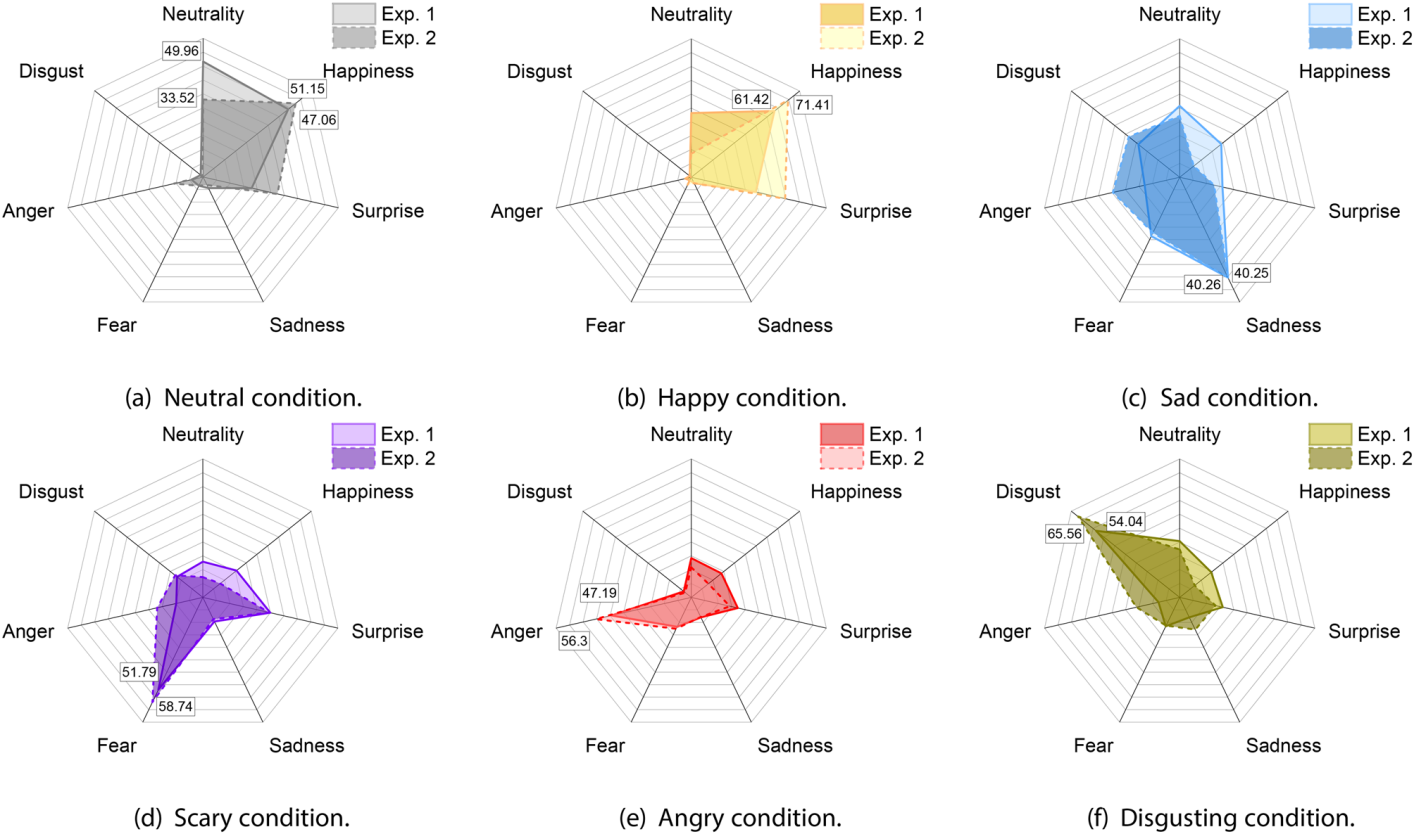
Comparison between the emotional rates in Exp. 1 and Exp. 2.

As suggested by Fig. 4, the two emotional labels which differed the most were Neutrality and Happiness. For Neutrality rates, we found differences between the two setups in the Neutral [*U*($N_{Exp.1}$ = 48; $N_{Exp.2}$ = 27) = 465; *Z* = − 2.03; *p* = 0.042)] (Fig. 4a), Happy [*U* = 348.5; *Z* = − 3.363; *p* $< 0.001$)] (Fig. 4b), and Scary [*U* = 453; *Z* = − 2.261; *p* = 0.023)] (Fig. 4d) conditions. For Happiness rates, the analysis highlighted differences between the two setups only for the Sad [*U* = 415; *Z* = − 2.714; *p* = 0.006)] (Fig. 4c), and the Disgusting [*U* = 466; *Z* = − 2.085; *p* = 0.037)] (Fig. 4f) conditions. In addition, Surprise rates differed only in the Happy condition [*U* = 443; *Z* = − 2.265; *p* = 0.023] (Fig. 4b). For the other environments participants’ rates were very similar and only Anger rates showed a small difference in the Happy condition [*U* = 465; *Z* = − 2.697; *p* = 0.005] (Fig. 4b).

Despite the few differences that emerged from the comparison, it is important to highlight that the overall trend followed by participants’ rates in our scenarios was consistent between the two samples. As visible from Tables 1 and 2, the comparisons for each label follow the same trend in both the experimental setups, except for Neutrality rates in the Neutral condition, which behave differently from Exp. 1 to Exp. 2.

#### Presence

Results did not show significant differences between the two experimental setups [Presence: *F*(1) = 1.108; *p* = 0.3; Involvement:*F*(1) = 0.147; *p* = 0.7; Sensory fidelity: *F*(1) = 0.534; *p* = 0.47; Adaptation/immersion: *F*(1) = 1.445; *p* = 0.23; Interface quality: *F*(1) = 0.494; *p* = 0.49].

## Discussion

This study tested ten new affective VEs categorised according to five emotional entities (i.e., happiness, sadness, fear, anger, disgust), plus a previously validated environment for neutrality^33^. The testing was carried out using two experimental setups, differentiated based on the technology used to reproduce the virtual environments. We then individually analysed the data collected in the two experimental setups and compared them to explore the reliability of virtual environments in arousing the same emotional response using different technologies.

To check possible biases in our participant sample, we used a validated scale (i.e. BEES) to assess the empathy capacity. Results showed a marginal effect of these measure on participants’ Sadness and Anger rates only in the Sad, Angry and Scary conditions.

Overall, the results confirm the effectiveness of our affective VEs in eliciting specific emotions, with a clear distinction between the scenarios related to Happiness, Anger, Fear, Sadness, and Disgust. The scenarios were rated significantly higher in the corresponding emotional label concerning all the other scales available. The environment used to elicit Neutrality received significantly higher rating in the corresponding label only in Exp. 1, while less defined results were obtained in Exp. 2, where we observed higher Happiness rates. Nevertheless, the Neutral environment was constituted by design elements shared with our Happy scenarios and this may explain the overlap obtained in our results.

Furthermore, the comparison between the two experimental setups confirms the high reliability and versatility of our environments in eliciting specific emotions even using different technological means to reproduce them. In fact, the results obtained in the two experiments are highly similar and present only a few exceptions, especially when considering the Neutral condition. For all the other emotional entities considered, the comparison showed the same trend shared between the two experimental setups and no statistically significant differences for the highest rated emotion in each condition, except for the Neutral scenario.

Regarding presence measures, we collected these data with two main objectives: (1) to compare if two different experimental setups with different levels of immersion provided different levels of presence; (2) to explore differences linked to the emotional component elicited in each environment. We did not find significant differences between the two setups, but results from Exp. 1 showed that our different conditions affected all the four factors constituting the Presence Questionnaire adapted for this study. The less involving scenarios were those meant to elicit Anger. Since the underlying mechanism followed in those scenarios was to frustrate users by continuously making them fail and restart, this is an expected and justified result. The most involving environments were those linked to Fear, opposed to the least involving scenario represented by the Neutral one, which also showed the lower level of Sensory Fidelity. However, this was an expected result since the constitutive items in this factor were mainly related to auditory features, which in this condition were voluntarily absent.

## Conclusions

Our initial validation promotes the possible application of the developed VEs in studies on emotions that involve the use of interactive and dynamic scenarios. Moreover, our results suggest the possibility to adapt hardware technologies and setup depending on the context and experiment requirements, without the risk of compromising the scenarios’ effectiveness. Therefore, these environments can be considered an initial step in developing a VR affective database capable of covering five distinct emotional entities. Future developments will require, to increase the level of immersion by using fully immersive technology such as HMD or CAVE, and to introduce a more detailed analysis of the emotional experience, for example, through the collection of physiological data and the analysis of facial expressions.
